# Evaluating the demographic and clinical minimum data sets of Iranian National Electronic Health Record

**DOI:** 10.1186/s12913-019-4284-x

**Published:** 2019-07-04

**Authors:** Reza Abbasi, Reza Khajouei, Moghadameh Mirzaee

**Affiliations:** 1 Health Information Management Research Center, Kashan University of Medical Sciences, Kashan, Iran; 2 Medical Informatics Research Center, Institute for Futures Studies in Health, Kerman University of Medical Sciences, Kerman, Iran; 3 Department of Health Information Sciences, Faculty of Management and Medical Information Sciences, Kerman University of Medical Sciences, Kerman, Iran; 4 Department of Biostatistics and Epidemiology, School of Public Health, Kerman University of Medical Sciences, Kerman, Iran

**Keywords:** Evaluating, Electronic health record, SEPAS, Minimum data set

## Abstract

**Background:**

Designing a standard data set is necessary to overcome the dispersion of data among different health information systems. The objective of this study was to evaluate the current demographic and clinical minimum data sets (MDSs) of Iranian National Electronic Health Record (known as SEPAS) and to identify most necessary data elements.

**Methods:**

Data were collected using a list of current demographic and clinical data of SEPAS and a self-administered questionnaire. All faculty members of six health related fields and the hospital authorities, and IT and HIM administrators of 6 hospitals in Kerman University of Medical Sciences were invited to participate in this study. The content validity of the questionnaire was confirmed by six medical informatics and HIM experts and the reliability was determined by Cronbach’s alpha (α =0.95). SPSS v18 was used to generate descriptive statistics**.**

**Results:**

Survey results indicated that 15 data elements should become mandatory elements of MDS for communicating data to SEPAS. These elements include patient’s name, surname, father’s name, nationality, cell number, job, residential address, residence place, passport number (for non-Iranian patients), diagnosis date, death time, death place and the unit of the hospital where the patient died. Moreover, participants suggested 33 additional demographic and clinical data elements to be communicated mandatorily to SEPAS.

**Conclusion:**

The results of this study showed that the minimum data sets of Iranian national electronic health record needs to be revised. Using the proposed MDSs by this study can improve the quality and efficiency of information and reduce redundancy by adding necessary data and preventing communication of unnecessary data. The method employed in this study can be used for investigating, refining and completing the MDSs of other health information systems.

## Background

Electronic health record (EHR) is one of the technologies that improves efficiency, management of health information, patient safety, and quality of health care by providing required health information to health care providers and policy makers [1]. According to world health organization (WHO) definition, EHRs provide access to lifelong health information of patients concerning their inpatient, outpatient, and emergency encounters [2]. Today, EHR has become one of the main information systems in healthcare centers and has replaced paper-based systems. Valuable information stored in EHR can be communicated and distributed across various information systems [3, 4].

Standardization of EHR data content facilitates sharing and exchanging information with other systems [3]. In any patient encounter, a large volume of data is generated and stored in EHR by different health care providers [5]. Reusing these data depends on many factors such as data quality, appropriateness for medical or research purposes [6]. Institute of Medicine (IOM) emphasizes on the importance of systematic, structured, and comprehensive data sets for analyzing health status of people [7].

Today, EHR is one of the technologies that have received much attention in the health domain. In Iran, the notion of electronic health was formed in 2001. Subsequently, the national electronic health record in Iran (SEPAS) and the health smart cards for whole Iranian population project were introduced in 2007 [8]. SEPAS is a national electronic health record for all Iranian citizens that collects patients’ data from all hospitals in a centralized system.

In the current phase of implementation, in order to aggregate inpatient health information, a number of demographic, clinical, and financial-administrative-insurance data elements, (optional or mandatory) called “documents cover”, are optionally or mandatorily sent from hospital information systems to SEPAS. A reason for optional communication of some data elements is the diversity of data collected for a patient in different hospitals due to the lack of a unique minimum data set in all 30 types of HIS used in Iran. Hence, full implementation of EHR can be achieved after resolving such problems [9]. Many hospital information systems (HIS) in Iran collect patients’ data in every patient encounter and communicate these data to SEPAS. Since these HISs do not use a unified minimum data set, collecting all required information in SEPAS is impossible. To overcome this problem, it is necessary to determine a set of standard data to be transferred from HISs to SEPAS.

Accurate and timely data improve policy making in health care [10]. Given the important role of information systems in health and treatment processes, they support successful decision making and implementing quality management in health care institutions [10]. Designing a standard data set is necessary to overcome the dispersion of data among different health information systems and enhancing the quality of data documentation. Moreover, interoperability of data among information systems needs a standard data set [11]. Defining minimum data set improves efficiency, effectiveness and accessibility of information contained in health information systems [12]. Likewise, using logical and appropriate data elements promotes information quality in EHR [4].

Because of the variation in data communication from health information systems to the national electronic health record, defining a standard data set can facilitate data communication and improve consistency [13]. Few papers have specifically reported on the design of minimum data sets for exchanging data from HISs to an EHR [14, 15]. Other previous studies have reported on the design of nursing minimum data sets for electronic health record [16–20] and MDSs for EHR subsystems such as radiology, laboratory and echography reporting systems [21–23]. Minimum data set is a standard method for collecting main data elements that helps to understand, interpret, compare, and report a large volume of data [10, 24]. This data set provides a framework for accessibility to accurate health information of patients, their demographic and clinical data, and their care plan. Moreover, it supports effective communication between healthcare providers and decision makers [25, 26].

Data elements in SEPAS are categorized into two groups; optional and mandatory. Mandatory data element refers to data items such as national ID codes, final diagnosis, and cause of death that should necessarily be sent from HISs to SEPAS. Among these elements national ID is the unique identifier for identifying each patient’s information. If one of these elements is incomplete, no data from a patient record will be sent to SEPAS. Optional data element refers to items, such as patient name, surname, father’s name, telephone number and patient’s address which are not obligatory. Without these items, other data elements of a patient record are sent to SEPAS.

Based on the investigations of health information management and medical informatics specialists, some of the data elements that are currently sent to SEPAS are unnecessary while a number of important data elements have never been sent to SEPAS. Thus, the aim of this study was to evaluate the current demographic and clinical minimum data sets of the national electronic health record in Iran and to design and propose standardized demographic and clinical minimum data sets. This study is a first step towards determining which demographic and clinical data elements should be collected in Iranian national electronic health records.

## Methods

### Study design and setting

This study was conducted in 2016. All specialists from six health-related fields (medical informatics, health information management, health services management, health policy, public health and epidemiology); and hospital directors, managers, and IT and HIM administrators of six hospitals affiliated with Kerman University of Medical Sciences were invited to participate in this study. Kerman University of Medical Sciences is one of the largest medical universities located in southeast of Iran.

### Exclusion and inclusion criteria

The participants were included if they had a direct or indirect role in policymaking, health planning or documenting and exchanging data to Iranian national electronic health record from this university and its affiliated hospitals and if they had at least 2 years of work experience in this university.

### Dataflow from HISs to SEPAS-patient

patients’ information is transferred from HIS to SEPAS on each patient encounter. Data elements submitted to SEPAS fall in two categories: mandatory and optional data elements. Mandatory demographic and clinical data elements such as national code for Iranian patients (as a unique identifier), final diagnosis and the cause of death should be completed necessarily. Otherwise, no patient information would be transferred. Transferring optional data elements is noncompulsory [9].

This study was conducted in two steps:

1. To evaluate the minimum data sets of SEPAS, we developed a list based on the instruction of Ministry of Health about the current core data that should be transferred from HISs to SEPAS [27]. Since mandatory data elements sending to Iranian national EHR, so we addressed to the necessity of the optional data element in order to sending to this EHR. The list consisted of 24 optional data elements (demographic and clinical) in SEPAS. The participants were asked to mention their view about the necessity of sending this data to SEPAS as mandatory elements.
2. Based on informal investigations and reports, we felt a gap between what is sent and what should be sent to SEPAS, so that a number of important data elements have never been sent to SEPAS. This reflected the need to a complementary minimum data set for SEPAS. Thus, we developed a self-administered questionnaire based on the information drawn from medical record forms used in hospitals and consensus of four medical informatics specialists (two with a medicine background, and two with health information management background) who had at least 5 years of work experience in hospitals. Questions in this step were about other demographic and clinical data elements of patient records that are not currently transferred to SEPAS. We first extracted 61 complementary data elements (14 demographic and 47 clinical elements) from patients’ records. Then we sought participants’ perspective about the necessity of adding these elements to the minimum data set of SEPAS.

These questions could be answered by selecting one on the four unnecessary, no idea, optional, and mandatory options. In order to obtain participants’ comments and recommendations, two open-ended questions were located at the end of each section.

Two health information management and four medical informatics experts (two with a medicine background, and two with health information management background) confirmed the content validity of the questionnaire. The reliability of the questionnaire was calculated Cronbach’s alpha (α =0.95).

One of the researchers distributed the questionnaires among the participants at their work places and provided sufficient instructions on how to complete the questionnaires. SPSS v18 was used to generate descriptive statistics**.** We categorized each data element as mandatory, optional, or unnecessary if more than 50% of the participants selected those options.

## Result

In total, 35 experts responded to the list and questionnaire. These participants consisted of five faculty members of medical informatics, two faculty members of health information management, three faculty members of epidemiology, two faculty members of public health, two faculty members of health services management and health policy, and four hospital directors, five managers, six IT administrators and six HIM administrators.

Table 1 shows the participants’ perspectives about necessity of sending the optional demographic and clinical data elements to SEPAS. About 19–31 participants (54–89%) voted to the mandatory communication of the following 15 demographic and clinical data elements, which are already optional, to SEPAS: patient name, surname, father’s name, nationality, cell and home telephone number, job, address, residence place, passport number (for non-Iranian patients), diagnosis date, diagnosis time, death time, death place and the unit of hospital where the patient died.

**Table 1:** Participants’ perspective about the necessity of sending the current optional demographic and clinical data elements to SEPAS

SEPAS data elements (All of this DEs is optional currently)	SEPAS mandatory marker	SEPAS optional marker	Survey > 50% participants identified as mandatory	Survey > 50% participants identified as optional	Survey > 50% participants identified as unnecessary
Patient name	–	✓	✓	–	–
Surname	–	✓	✓	–	–
Father’s name	–	✓	✓	–	–
Father’s surname	–	✓	–	–	–
Mother’s name	–	✓	–	–	–
Mother’s surname	–	✓	–	–	✓
Nationality	–	✓	✓	–	–
Birth Cert. number	–	✓	–	–	–
Patient address	–	✓	✓	–	–
Zip code	–	✓		–	–
Home telephone number	–	✓	✓	–	–
Cell number	–	✓	–	–	–
Educational level	–	✓	–	–	–
Patient job	–	✓	✓	–	–
Patient job description	–	✓	–	–	–
Patient residence place	–	✓	✓	–	–
Birth Cert. issuing place	–	✓	–	–	–
Passport number (for non-Iranian patients)	–	✓	✓	–	–
Diagnosis date	–	✓	✓	–	–
Diagnosis time	–	✓	✓	–	–
Diagnosis severity	–	✓	–	–	–
Death time	–	✓	✓	–	–
Death location	–	✓	✓	–	–
Unit of hospital where patient died	–	✓	✓	–	–

Table 2 presents the perspective of participants about necessity of recording and communicating complementary data elements to SEPAS. About 21–25 participants (60–71%) agreed to the mandatory communication of complementary data elements such as unit number, age, province and city and the deceased person’s full address to SEPAS.

**Table 2:** Participants’ perspective about the necessity of recording and communicating complementary data to SEPAS

		Survey > 50% participants identified as mandatory	Survey > 50% participants identified as optional	Survey > 50% participants identified as unnecessary
Demographic data	Patient unit number	✓	–	–
	Nickname	–	–	✓
	Age	✓	–	–
	Province	✓	–	–
	City	✓	–	–
	Driver’s license number	–	–	✓
	Military status	–	–	✓
	Religion	–	–	–
	Denominations	–	–	–
	Ethnicity	–	–	–
	Dialect	–	–	✓
	Spouse personal details	–	–	–
	Death certificate number	–	–	–
	the deceased person’s full address	✓	–	–
Delivery and childbirth	Type of delivery	✓	–	–
	Cause of delivery	–	–	–
	Delivery location	–	–	–
	Number of newborns	✓	–	–
	Birth order	✓	–	–
	Newborn weight	✓	–	–
	Newborn Health status	✓	–	–
	Congenital anomalies	✓	–	–
	Newborn unit number	✓	–	–
Patient examinations	Main complaints	✓	–	–
	Primary diagnosis	✓	–	–
	Diagnosis during treatment	✓	–	–
	Physician orders	✓	–	–
	Physical examination and clinical investigation	–	–	–
	Nurse observations	–	–	–
	Underlying disease	✓	–	–
	Family history	✓	–	–
Vital signs	Systolic blood pressure	✓	–	–
	Diastolic blood pressure	✓	–	–
	Heart rate	–	–	–
	Respiratory rate	–	–	–
	Temperature	–	–	–
Operations	Operation name	✓	–	–
	Type of operation (outpatient, inpatient)	✓	–	–
	Date of operation	✓	–	–
Anesthesia Allergies	Type of anesthesia	✓	–	–
	Anesthetics	–	–	–
	Anesthesia time	–	–	–
	Type of allergy	✓	–	–
	Allergens	✓	–	–
	Severity of allergy diagnosis	–	–	–
	Date identify allergies	–	–	–
Specific patient conditions	Pregnancy or breastfeeding	✓	–	–
	Alcohol consumption	–	–	–
	Smoking	–	–	–
	Tobacco usage	–	–	–
	Prosthesis in patient body	✓	–	–
	Diet	–	–	–
Medications	Medication name	✓	–	–
	Medication type (Therapeutic)	–	–	–
	Medication form	–	–	–
	Medication dose	–	–	–
	Medication usage	–	–	–
	Patient medication history	✓	–	–
	Drug sensitivity	✓	–	–
Blood type	Blood group	✓	–	–
	Rh	✓	–	–

Currently data elements related to delivery and childbirth are not collected in SEPAS. The investigation of participants’ perspective about the necessity of recording and communicating these elements to SEPAS revealed that approximately 19–29 participants (54–83%) demanded adding the following elements: type of delivery, newborn unit number, newborn health status, newborn congenital anomalies, the number of delivered newborns, birth order, and newborn weight.

About 18–32 participants (51–91%) recommended the following data elements to be recorded and communicated to the SEPAS main complaint, primary diagnosis, diagnosis during treatment, physician orders, underlying disease, family history, systolic and diastolic blood pressure, operation name, type and date, anesthesia type, allergies, allergen factors, patients’ specific conditions such as pregnancy or lactation, as well as the existence of artificial limbs in the patient body, medications, patient medication history and allergies and finally blood group and Rh.

Totally, four participants responded to open-ended questions. According to the results of these questions, two participants proposed complementary elements such as external causes of injury, location of accident (province, city, village, street, etc.), physical impairment, and patient’s specific conditions including his disability and limitations to be communicated to SEPAS as optional elements. Three other participants proposed using unique numbers such as national ID, health insurance number, or medical record number to be able access all demographic and clinical data of a patient. Also it prevents communicating a large amount of data on each patient encounter.

## Discussion

Participants, in this study, believed that less than two-thirds of the optional data elements should be mandatorily sent to the national electronic health record. As well, they proposed 33 complementary data elements (5 demographic and 28 clinical DEs) to be added to the current data set of EHR. Also, the participants expressed that communicating data elements such as father’s and mother’s surnames and patient job description that are currently sent to the national EHR, is unnecessary.

According to the results of this study, recording and communicating patient name, surname, father’s name, age, nationality, passport number (for non-Iranian patients), cell and home telephone number, job, province, city and residential address to SEPAS should be mandatory (currently these DEs are optional). The results of other studies [14, 28–30] showed that collecting demographic and identification information such as name, surname, age, address and patients mobile number in EHR is necessary. The resulted data elements in our study are largely similar to those of previous studies. However, none of these studies addressed the job, nationality, and father and mother’s names of patients in their data sets. According to WHO guidelines about medical records and electronic health records [2, 31], ideally, to identify a person when a national identification number is not used, institutions need to determine what piece of information is not likely to be changed. Some countries use father’s or mother’s names, biometric characteristics or personal national insurance number or social security number. Consistent with this, participants in the current study believed that patient father’s name should be collected and communicated to the national electronic health record, but patient mother’s name is not required.

The participants of our study demanded that delivery and the newborn baby data elements such as delivery type, newborn unit number, newborn health status, abnormalities, newborn number and order in terms of birth, and newborn weight should be added and sent to SEPAS. Since, people’s health information from birth to death is stored in EHR [2], it is necessary to pay due attention to gathering people information at birth. Mother and their newborns are a vulnerable group of population [32]. Annually, more than 10 million perinatal deaths occur in the world [33], which makes delivery and perinatal data important indices of health. In order to enhance the quality of these data and to facilitate data collection, developing newborn, MDS is highly recommended [34]. In many countries like Australia, MDS for newborn babies is designed to achieve epidemiological purposes, promote health status of mothers and newborns, identify and reduce congenital anomalies, evaluate health care of newborns, compose statistical reports and indices, and finally to make the national health policies [34]. Therefore, adding and communicating newborn data elements proposed in this study to the national electronic health record can contribute to the success of electronic health record and attaining macro-goals in health area. To our knowledge, this is the first study that proposed a neonatal minimum data set for communication of data from hospital information systems to an EHR. Other studies only suggested perinatal minimum data set to collect standard national perinatal data and retrieve neonatal outcomes information [35, 36]. During the treatment process, it is highly important for physicians to access medical history, clinical examinations, physical observation and disease symptoms of patients. Accessibility of patients’ medical history from the early stage of encounter can reduce medical errors and medication side-effects [37]. Based on participants’ perspective, a number of clinical data elements such as main complaint, primary diagnosis, diagnosis during treatment, physician orders, and patient’s and family history must be recorded and communicated to the national electronic health record. These findings are consistent with the results of previous studies [14, 30, 38–40]. Although these studies proposed minimum data sets similar to our results, collecting these data in Iran is mostly limited to the paper and electronic records of patients in hospitals, and are not recorded and communicated to SEPAS. Participants in this study believed that underlying disease data elements should be recorded and communicated to the electronic health record, while; previous studies did not investigate this issue.

Disease symptoms, vital signs, blood group and Rh, operation, medication information, and patient allergy are other elements that participants rated as mandatory to be sent to the national electronic health record. Similarly, Latha and Hayrides [14, 30] emphasized on the importance of these elements in the electronic health records and cards. Our study showed that main operation and anesthesia information such as operation name, type, and date, as well as, anesthesia type were other data elements that should be recorded and communicated to EHR. Although previous studies also addressed the necessity of collecting operation data elements [14, 30] and patients’ vital signs [14, 40] in health information systems, they did not address the communication of anesthesia information to EHR.

Blood group and Rh of patients are important elements that do not exist in the national electronic health record and many HISs in Iran. In line with the results of other studies on EHR and smart cards [14, 29, 30], participants in this study believed that information about blood group and Rh should be recorded in the health information systems and EHRs.

In accordance with our results, other studies [14, 28, 40, 41] emphasized the necessity of collecting data elements related to patients’ allergies. Results of this study, identified information concerning specific patient conditions such as pregnancy, lactation and existence of artificial limbs in patient body as mandatory data elements while, information about patient diet was identified as an unnecessary element. Contrary to these findings, the results of Abdolkhani’s study [38] showed that diet and food allergies should be available in athletes EHR. A reason for this inconsistency is that the diet of all athletes should be monitored routinely regardless of their health condition. While this issue is only important for a limited number of patients such as patients with diabetes or hypertriglyceridemia.

Based on the results of this study and other similar studies on the different health information systems [28, 38, 40] and electronic health record in Finland [14], we propose adding medication name, medication history and side-effects, drug allergy, type of allergy, and allergen’s name data elements to EHR minimum data set. Medication errors are among common medical errors imposing heavy costs on health care centers. Availability of patient drug allergies in EHR can save patients’ lives in emergency situations [42, 43] and reduce medication errors and even deaths [44, 45]. According to Bates’s study, [44] 12.9% of medication errors occur when drug allergy information is not documented.

Based on our findings, information on patients’ death including death time and location, unit of hospital where patient died (currently these DEs are optional), cause of death and the deceased full address should be sent to electronic health record. Porock [46] in designing minimum data sets of mortality risk index, categorized MDSs about dead patients in 4 categories: demographic data, disease information such as disease type, information about signs and symptoms of disease and also the cause of death and its side-effects. However, Porock’s study did not indicate the necessity of elements such as time and place of death and the unit of hospital where the patient died. Information about time and place of death mostly are used in legal cases by forensic medical centers and other judicial institutions. Information on the unit of hospital where a patient died along with cause of death can be used for identifying high-risk units and for making preventive policies to reduce mortality.

Collecting structured and standardized data is a way to enhance quality of data documentation [11]. Automation of data collection and storage system and procedures promulgated the use of minimum data sets [47]. MDSs can improve the quality of service and provide important data to support cross-country planning and decision making in health care [15, 48, 49]. Minimum data sets often include two groups of demographic and clinical data [50]. Demographic data are important for patients’ identification, determining the distribution of age and gender, and communication with patients [51, 52]. Clinical data are used in diagnostic and treatment process and to help health care research, planning, and policy making [51].

Based on a standard developed by the Royal Australian College of General Practitioners [53], EHR should contain information about patient’s history, health status, clinical procedures, medication usage and administrative work. Promoting information quality in EHR requires documenting necessary data about family and social history, medication usage, smoking and blood pressure, consultations, reasons of health care encounters, clinical findings and other information that facilitate clinical decision making and providing appropriate health care services [53].

There are a number of challenges for EHR implementation and a long way to its meaningful use in many countries [54]. The forecasting of a study showed given that there are no major policy changes, the maturity of EHR (movement from a paper based environment to a fully electronic environment) in the most US hospitals may take up to 2035 [55]. Managers and policy makers require an accurate assessment of the EHR implementation challenges in order to design an effective program for implementation of health information systems [54].

Collecting standard and required data from diverse health information systems can help to create more comprehensive health records such as population health record (PopHR) and community health record (CHR). These records can provide a comprehensive vision of population health status and the factors that influence it by exchanging or receiving data from many national data sets and systems such as HISs and EHR. Moreover they can be used by public health agencies in each country [56, 57].

To our knowledge, this is the first study evaluating the inclusion of different demographic and clinical data elements in an EHR minimum dataset. Besides the data elements addressed by other studies, this study also investigated the necessity of recording and sending patient job, nationality, father’s and mother’s name of patients, anesthesia information, underlying disease, time and place of death, the unit of hospital where the patient died and newborn information from HISs to EHR.

This study sought the perspective of different groups of experts from different fields of health such as medicine, epidemiology, public health, health services management, health policy, medical informatics and health information management specialists as well as hospital directors, managers and IT and HIM administrators of educational hospitals in one of the top universities in Iran. These participants are directly and indirectly engaged with patients and their information.

### Limitations and future recommendations

This study had three limitations. First, we did not seek the reasons for selecting every specific element by participants. Due to the long list of elements this could affect the participation and the response rate. However, the data related to participants’ perspectives were analyzed cumulatively. Second, although a ‘self-administered’ questionnaire would help rank the variables, it may not be a sufficient method to decide on the relevance of a specific data element. Thus, recruiting more participants from other universities or employing other methods like Delphi technique or focus group discussion may yield more accurate results. However, this study evaluated the data elements of an electronic health record and proposed a refined version by adding complementary data elements. Using these results can help better implementation of EHR in Iran and improves information exchange from hospital information systems to EHR. Third, although developing the required minimum data sets is one of the main steps toward standardization of data exchange among health information systems, to achieve a high level of standardization other factors such as using common terminology, data structure, and data exchange protocols should also be addressed. We suggest that future similar studies address these factors too.

### Implications for research and practice

The results of this study showed that about one-third of the data communicated daily from hospital information systems to the electronic health records is unnecessary. Moreover, this study identified a number of necessary data elements that are not communicated to EHRs. The results of this study can help to enhance the accuracy and completeness of required data and to prevent data redundancy. Health care authorities, policy makers, and administrators can utilize these results in order to enhance effectiveness of health services by providing relevant, complete and accurate information to providers at the point of care. The results of this study can be used by designers and developers of health information systems and EHR for developing new systems or upgrading current systems. This study presented a method to enhance the quality of clinical and administrative data collection and storage in universities and ministry of health databases.

## Conclusion

The results of this study shed light on miscommunication of a number of clinical and administrative data elements to electronic health records. Based on these results, some necessary data elements are not recorded and communicated but some other unnecessary element are communicated. Lack of information such as clinical history and examination, reasons for the visits and family history can affect diagnostic process. Missing information related to operation, anesthesia, medications, allergies, specific conditions of patients and information about blood group and Rh compromises prevention and treatment processes of patients.

Since, one of the main purposes of EHR is providing access to health and treatment information of people, poor documentation of necessary information may adversely affect clinical decision-making, planning and policy-making in health care domain. This study was as a first step towards determining which data elements should be collected in Iranian national electronic health records by refining the current data sets. Collecting unnecessary data in an EHR leads to data redundancy, also, failure to send necessary data can reduce the quality of collected data. The method employed in this study can be used by EHR developers and policy-makers to improve the quality of data collection and communication.

## Data Availability

Since our study is a descriptive study, all data represented in the tables.

## Abbreviations

CHR: Community Health Record
EHR: Electronic Health Record
MDS: Minimum Data Set
PopHR: Population Health Record
SEPAS: Iranian National Electronic Health Record
